# Inference of sparse combinatorial-control networks from gene-expression data: a message passing approach

**DOI:** 10.1186/1471-2105-11-355

**Published:** 2010-06-29

**Authors:** Marc Bailly-Bechet, Alfredo Braunstein, Andrea Pagnani, Martin Weigt, Riccardo Zecchina

**Affiliations:** 1 Université Lyon 1; CNRS UMR 5558, Laboratoire de Biométrie et Biologie Evolutive, F-69622, Villeurbanne, France; 2 Politecnico di Torino, C.so Duca degli Abruzzi 24, I-10129 Torino, Italy; 3 Human Genetics Foundation, Via Nizza 230, I-10126 Torino, Italy; 4 ISI Foundation Viale Settimio Severo 65, Villa Gualino, I-10133 Torino, Italy

## Abstract

**Background:**

Transcriptional gene regulation is one of the most important mechanisms in controlling many essential cellular processes, including cell development, cell-cycle control, and the cellular response to variations in environmental conditions. Genes are regulated by transcription factors and other genes/proteins via a complex interconnection network. Such regulatory links may be predicted using microarray expression data, but most regulation models suppose transcription factor independence, which leads to spurious links when many genes have highly correlated expression levels.

**Results:**

We propose a new algorithm to infer combinatorial control networks from gene-expression data. Based on a simple model of combinatorial gene regulation, it includes a message-passing approach which avoids explicit sampling over putative gene-regulatory networks. This algorithm is shown to recover the structure of a simple artificial cell-cycle network model for baker's yeast. It is then applied to a large-scale yeast gene expression dataset in order to identify combinatorial regulations, and to a data set of direct medical interest, namely the Pleiotropic Drug Resistance (PDR) network.

**Conclusions:**

The algorithm we designed is able to recover biologically meaningful interactions, as shown by recent experimental results [1]. Moreover, new cases of combinatorial control are predicted, showing how simple models taking this phenomenon into account can lead to informative predictions and allow to extract more putative regulatory interactions from microarray databases.

## Background

Transcriptional gene regulation is one of the key mechanisms in living cells; the control of gene expression is crucial in processes as cell development, cell-cycle regulation, and response to external stimuli [2-5]. While the number of sequenced genomes is growing rapidly, it becomes more and more important to study genetic information on a higher level, *i.e*. to understand genes in their interdependence and to capture relations between regulatory genes, e.g. transcription factors (TF) or signaling proteins, and regulated genes via the reconstruction of gene-regulatory networks (GRN).

Direct experimental approaches to understand gene regulation are money and time consuming. Therefore genome-scale regulatory networks are only known for *E. coli *[6] and for baker's yeast, *S. cerevisiae *[7,8]. For higher organisms, the knowledge is restricted to intensively studied small functional modules, see e.g. [9,10]. Some characteristic features of these GRN are:

• *Directionality*: Regulatory control is directed from regulators to regulated genes.

• *Sparsity*: Each single gene is controlled by a limited number of other genes, which is small compared to the total gene content (and also to the total number of TFs) of an organism.

• *Combinatorial control*: The expression of a gene may depend on the joint activity of various regulatory proteins.

The last item is crucial, and it is the topic of very active and diversified research [11-15]. One example of combinatorial control in yeast is the case of transcription factors Yrr1 and Yrm1, which compete for occupancy of the same promoter sequence [16]. Many other types of combined control exist, such as the formation of hetero- or homo-dimers by TFs, or their post-translational modification by other proteins, which can entirely change their targets [17]. On the other hand, the hypothesis of sparsity has been experimentally checked in well-studied organisms, where it has been observed that the number of TFs is low compared to the total number of genes.

It is tempting to ask in how far GRN can be reconstructed from gene-expression data. After the advent of the first generation of gene-expression microarrays, more than a decade ago [18], we face an growing number of new high-throughput technologies capable of monitoring simultaneous concentrations of thousands of cellular components, in particular of mRNAs. The improved quality of new generations of microarrays, the decrease of their cost, and the amount of experiments accumulated so far call for the development of large-scale methods of data analysis. Different approaches to modeling have been proposed (see [19] for a recent review), from a coarse-grained description of co-regulated genes [20], classification methods [21,22], to Boolean descriptions where genes are described in terms of logical switches with only *on/off *states of activity [23] (and in particular [24] for the problem of inference of boolean networks), or considering more realistic systems of differential equations describing the kinetic details [25]. Also for GRN reconstruction, approaches from different origins have been proposed: system control theory [26-29], Bayesian inference [30-33], information theory [34-36].

Many limitations of the existing algorithms arise directly from the quantity and quality of data:

Microarrays are noisy averages over cell populations, and the number of available arrays is normally much smaller than the number of probes measured in each array. Moreover, microarrays measure mRNA but not active protein concentrations (which, for TFs, are the important parameters). Both may be uncorrelated in the cell [37]. But as proteomics data are even sparser than microarray data, this is not an easy-to-solve problem, and many modeling approaches use mRNA concentration alone. Another problem is the existence of combinatorial control in gene regulation: Predicting such cases is a NP-complete problem, and has therefore eluded many approaches due to computational complexity, although some recent and interesting progress has been achieved in [33].

In this paper we introduce a novel algorithmic strategy, based on message-passing techniques, to infer the regulatory network of an organism based solely on genome-wide expression data, that specifically focuses on combinatorial control. Our methodology is probabilistic and distributed, allowing for a fast exploration of the space of networks. We apply the algorithm to three yeast networks: (i) To test the efficiency of the algorithm, we first reconstruct an *in-silico *regulatory network for cell-cycle control from artificially generated data [38]. (ii) We propose a large-scale reconstruction of the yeast regulatory network, using the classic Gasch microarray dataset [4], and analyze evidence for combinatorial control. (iii) We use yeast expression data from the SMD database [39] to recover the regulations affecting genes involved in pleiotropic drug resistance (PDR). This network is now under intense scrutiny because of the more and more common nosocomial infections by *Candida *yeasts [40], which are able to resist to drugs by exporting them out of the cell. These resistance mechanisms are genetically regulated by the PDR network, which we aim to reconstruct. An detailed description of the algorithm is given in the Methods section. An implementation in C can be downloaded at [41].

## Results and Discussion

### Reconstructing an *in-silico *yeast cell-cycle network

Before coming to biological data, we test our approach on the network model of Tang *et al. *[38] for cell cycle regulation in *S. cerevisiae*. The cell cycle is regulated by cyclins/CDK complexes, which sequentially activate and inhibit each other, creating a periodicity which is the clock of the cell. Recently sequential waves of transcriptional activation independent of cyclins activation have been discovered [42,43], but they are not taken into account in the model. It anyway serves as an ideal starting point for the the performance analysis of our analysis, since the data generating network is explicitly known and can be compared to our inferred regulatory interactions.

In the model of [38], the regulatory network consists of *N *= 11 genes/proteins, which are described by a binary state vector . Interactions are encoded into a coupling matrix  with entries  in total the model of [38] contains 15 activating and 19 repressing links. The definition of the network model is completed by the dynamical rule(1)

with(2)

Our aim here is to infer the regulatory links of this network model based on the different state vectors s^*t*^. The above *in-silico *dynamics shows 7 fixed points, *i.e*. stationary states of the dynamics. Each fixed point can be characterized by the size of its basin of attraction, *i.e*. by the number of initial random initial conditions that end on it. Tang *et al. *argue that the fixed point with the largest basin of attraction can be identified with the *G*1 phase of the cell cycle. If one perturbs the stationary *G*1 state by flipping the Cln3 cyclin to its active value, the network passes trough 13 different states before reaching again *G*_1_. The authors of [38] argue that this trajectory robustly reproduces various aspects of the yeast cell cycle.

We test our algorithm on two different data sets: (i) the 13 states obtained by first flipping the Cln3 cyclin to the active value, and letting the system evolve until stationarity as described before, (ii) a larger dataset containing the configurations of data set (i) and additionally the trajectories obtained by evolving all configurations at Hamming distance 1 away from *G*_1 _(70 different states). In Additional File 1 we include both data sets together with the links of the network.

In order to deal with time series, Eq. (9) for the prior probability distribution is transformed into , to express the conditional probability of the target gene 0 at time *t *+ 1 given the expression profile of the other genes at time *t*. For both data sets we fix the diluting field *h *to a value giving N_eff _~ 30 according to Eq. (5). For the original data set (i) we fix σ_*D *_0. while for the larger data set (ii), convergence of Belief propagation (BP) is ensured by σ_*D *_= 0.3.

**Fig 1:**
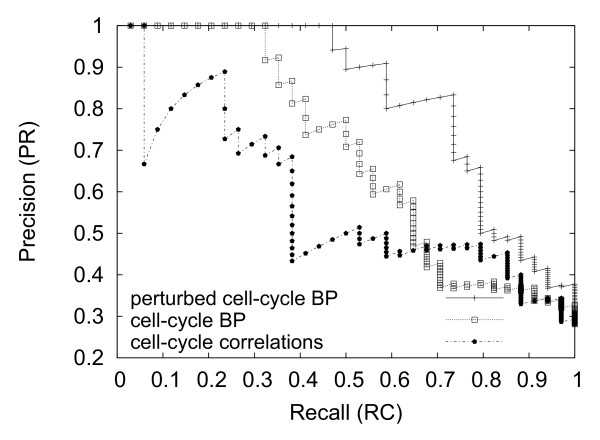
**Precision-Recall curve for the cell-cycle inference**. *Precision-Recall *curve for the network inferred using BP (both cell-cycle and perturbed cell-cycle data sets) and Pearson correlation coefficient (only cell-cycle data set). In the case of BP-based inference we infer correctly 11 links (cell-cycle) and 16 links (perturbed cell-cycle) respectively before making the first error. In the case of the correlation based inference we make the first error after only 2 correct links.

In Fig 1 we display the *Precision-Recall *curve for the network inferred using BP, for both cell-cycle and perturbed cell-cycle data sets (cf. the paragraph about observables in Methods for a precise definition of precision and recall). Results are compared to the performance of a co-expression network which ranks links *j *→ *i *according to the Pearson correlation of  and . We see that on the original data set BP is able to correctly infer 11 links before making the first error, whereas Pearson correlation fails already after two correctly predicted links. This result shows that BP correctly manages to take into account combinatorial control effects, which cannot be seen by purely local methods (as pair correlations). Increasing the data set improves the outcome of BP, the larger data set leads to 16 correctly predicted links before the precision drops down from one, and the precision stays always above the one obtained from the 13-state trajectory. It is also interesting to note that the first links inferred by our algorithm are those which where identified in [44] as essential for reproducing the cell-cycle by a complete enumeration of the space of all networks.

### Yeast response to environmental stresses

For a second application of BP - at much larger scale - we use the data of Gasch *et al. *[4], which consist of 172 genome-wide microarrays of *S. cerevisiae *under different environmental conditions. We filter out all genes, which show little differential expression (variance smaller than three times the minimal variance measured) or which miss more than 10 data points. Thereby the gene number is reduced to 2659 target genes, *i.e*. to roughly half of the entire genome. As putative regulators we consider (i) genes annotated as transcription factors or structurally similar to known transcription factors, and (ii) genes involved in signaling [45]: their total number sums up to 460 putative inputs.

We run our algorithm with σ = 0.25 which equals the minimal variance of a gene found in the full data set. BP giving probabilistic results, we kept regulatory links with more than 95% of confidence.

As the distribution of the marginal probabilities follows a power-law distribution (data not shown), changing this threshold (e.g. going to 99% or 90%) has little effect on the final network. The network contains 5779 regulatory links, giving an average of 2.17 links per target; the in-connectivity has a distribution best fitted by an exponential law *k *= *Ce*^-γ ^with γ = 0.42, a value very close to the reference one in [7]. Only 182 target genes (7%) have no predicted regulator. Moreover, 1637 targets (62%) are regulated by at least 2 genes, providing a wealth of potential predictions in the field of combinatorial control. Interestingly enough the finding of 2.17 links per target can be confronted with the result of Balaji *et. al. *[46], based on a review of Chip-chip experiments, reporting a comparable average value of 2.9 regulators per target.

#### Combinatorial control

In order to assess the relevance of the inferred network, we compare it first to a network based on pairwise correlations of expression data (co-expression network), which was constructed to have the same number of links as the BP network. Selected links are those of highest absolute value of the Pearson correlation between all input-output gene pairs. This is clearly an oversimplified model, but it allows to grasp the significant features of our model.

One advantage of our algorithm is the explicit inference of combinatorial control mechanisms by multiple transcription factors. Indeed, the number of genes with multiple regulators inferred using our methodology is 1637, while it is only 612 in the case of the pairwise-correlation network. The average number of regulators per *regulated *gene (i.e. genes with at least one inferred regulator) in our BP case is 2.33, and has to be compared to 2.9 from the work of Balaji *et al. *[46], and 6.17 for the co-expression network. It is interesting to note that BP results are is closer to the experimental network as compared to the co-expression one. This feature shows how, for the vast majority of target genes, our algorithm is able to describe the behavior of the gene by combining few putative regulators.

Another way of investigating combinatorial control is to compare expression profiles of different regulators. Regulators having highly correlated expression profiles carry similar information to the target gene, whereas regulators having diverse profiles can be used to transmit much more information. This is directly incorporated in our model: The sparsity term introduced in Eq. 5 reduces the effect of potential regulators whose expression profiles are highly correlated. As a limiting example let us consider two input genes with identical expression profiles, regulating one target gene. The sparsity term will select randomly only one of the two, and identify it as a regulator. In more realistic cases, no two genes shows exactly the same expression, and only the most *explanatory *gene will be chosen as a regulator out of a set of highly correlated potential TFs.

**Fig 2:**
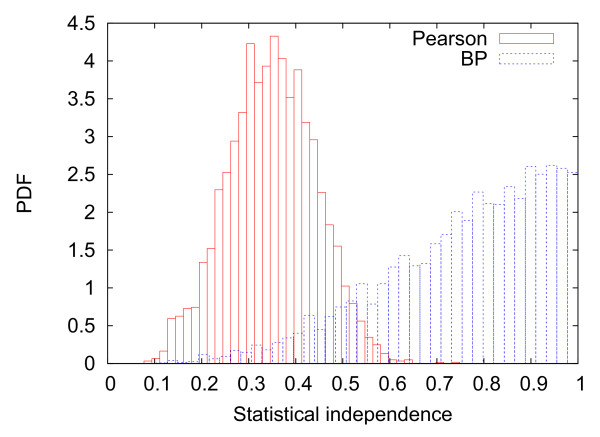
**Statistical independence Probability distribution**. Plot of the statistical independence probability distribution (*i.e*. 1-|Pearson correlation coefficient|) between pairs of TF regulating combinatorially the same target. Red curve: Co-expression network. Blue curve: BP network. Note that the statistical independence is much higher between TF inferred by BP, showing higher information content in combinatorial control.

**Fig 3:**
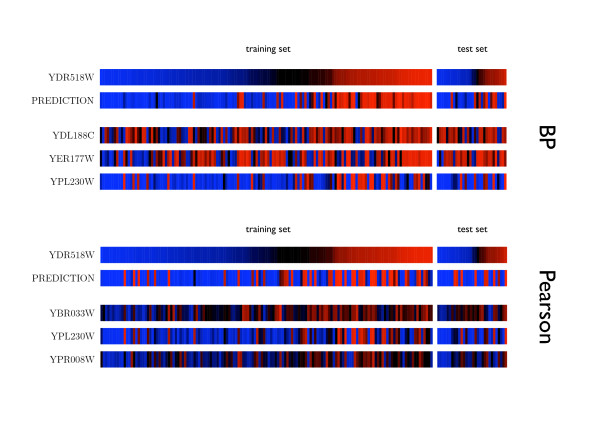
**An example of combinatorial control**. Example for combinatorial control. Top: Three top inputs found by BP, with the prediction compared to the real output. The left bars are training, the right test data. Bottom: Three most Pearson-correlated genes and corresponding prediction. Over- (under-) expression is depicted in red (blue).

To quantify the independent information carried by each regulator we compute, as a simple measure, one minus the Pearson correlation coefficient between any two regulators of common target genes, see Fig 2. One can see that the information content is much higher using our methodology than simply co-expression, because the latter tends to discover redundant information as displayed in the example of Fig 3 for the target gene *YDR518W*. This specific example also shows that secondary regulators found by BP tend to correct discrepancies between the first regulator and the target gene.

#### Comparison to experimental TF binding data

In order to further investigate the significance of the BP inferred network, we compare it to the experimentally verified network presented by Balaji *et. al. *[46], as characterized by 158 TFs, 4411 target genes, and 12974 regulatory links between them. After filtering out genes with low variance in the expression data set, the set of analyzed genes consists of 1919 targets, and 132 TFs. The number of experimentally verified links between these genes reduces consequently to 5533. Again we run BP with σ = 0.25, which equals the minimal variance of a gene found in the full data set, and we keep regulatory links with more than 90% of confidence. The resulting network has 6914 directed edges. Since these edges describe logical implications between gene expression levels, it is not clear in how far they reflect physical binding between the TF related to the input gene, and the promoter sequence of the target gene. It is easy to imagine that co-regulated genes are discovered as predicting each other, or secondary targets in regulatory cascades are recognized as direct targets.

In fact, the overlap with the experimentally verified network is only 206 edges (the resulting network is provided in Additional File 1). In order to give a statistical assessment of this number, we compare it to the overlap with a null model: We scramble the links in the BP network randomly preserving the in-degree of the inferred network. The overlap with the null model is 176 ± 5.3 edges, implying a z-score of 5.5, and a p-value of 1.18 × 10^-8 ^(under the hypothesis that the distribution of overlaps is Gaussian with mean and variance given by the null-model).

To check the effect of an increased number of experiments, we downloaded 1013 microarrays from the Stanford Microarray Database (SMD) [47]. Now 2614 target genes and 157 regulatory genes pass the statistical test, and the coverage of the experimental network increases to 7635 links. With respect to Gasch's data set, we use a 6-fold higher number of arrays coming from different experiments, so we run BP at a higher noise value σ = 1.5. The resulting BP network has 16176 edges (around three times the number of edges inferred with Gasch dataset alone). The overlap with the experimentally verified network is 406 edges (the resulting network is provided in Additional File 1). The overlap with the null-model is 314 ± 7.9 edges. Thus we find a z-score of 11.6, and a p-value of 1.6 × 10^-31^. As a comparison, we decided to analyze the same data set and the same set of 157 potential transcription factor with the ARACNe software [35]. To obtain statistically similar networks we set the *data processing inequality *threshold (a tunable parameter for controlling the overall number of edges in the network) to 0.10: the resulting network has 19775 directed edges (note that ARACNe produces undirected links). The overlap with the experimentally verified network is of 480 edges (data in Addition Files). The overlap with the null-mode is 424 ± 9.8 edges, with a z-score of 5.7 and a p-value of 3.0 × 10^-9^.

The sensible increase of statistical significance with respect to the results using Gasch's data is encouraging: It indicates in quantitative form, that larger microarray numbers would allow for extracting substantially more information about regulatory processes from gene expression data.

### Inference of the PDR network

**Fig 4:**
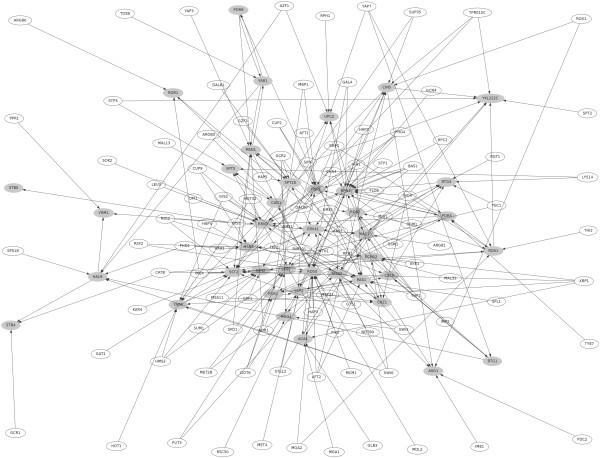
**The PDR network inferred**. The PDR regulatory network inferred by BP, comprising 157 TF and 40 targets. Targets are shown in grey.

We finally apply our algorithm to a small dataset, to tackle an issue of direct medical relevance: drug resistance among yeasts. *S. cerevisiae *is able to resist many drugs, using an ensemble of genes connected in the "pleiotropic drug resistance" network. The basic mechanism is that these genes, regulated by the master regulator PDR1, can export a broad range of substances out of the cell - drugs included. This general feature has been discovered in many organisms, and is considered a generic and robust mechanism of drug resistance, from bacteria to yeasts [48]. The precise regulations acting in this network are yet unknown, even if numerous works have already uncovered a part of them [49-52]. Here we propose to look for combinatorial regulations in this network, in order to better understand how transcription factors dedicated to drug resistance collaborate to ensure cell survival in harsh conditions - that is, in the presence of drugs. We run our algorithm on 40 genes known to be involved in PDR processes as targets - selection was based on literature -, and use all 157 transcription factors annotated in the database YEASTRACT [53] as potential regulators. The expression data consist of 912 microarrays from SMD [47]. Due to its small size, the statistical properties of the inferred network (see Fig 4) are quite different from the global one : 265 links were inferred at 95% confidence, giving a high average of 6.65 regulators per regulated gene. All target genes had at least one regulator; in fact only one had a single regulator (the GIS1 → STB5 couple).

Again, as a comparison, we decided to analyze the same data set ARACNe. To obtain statistically similar networks, we set the *data processing inequality *to 0.10: 247 links were inferred (note that ARACNe produces undirected links). Both networks are provided in Additional File 1. As a first observation we note that 13 out of the 40 target gene appear not regulated in the ARACNe network. We can conclude that, at least in this case, ARACNe seems to produce links which are more *concentrated *to a smaller target number, with an in-degree of 9.14 ± 6.6 TF/regulated target (to be confronted with the BP results of 6.625 ± 3.6).

Compared to the latest version of YEASTRACT, we find the following numbers of overlapping links: 16 in our case (if we consider the TF → target direction), and 28 if the direction is not taken into account. ARACNe, which produces an undirected network, has only 22 overlapping links. We also compared our findings with the network presented in the work of Balaji *et al. *[46]: in the BP case we match 8 directed edges and 15 undirected ones, whereas ARACNe matches 9 undirected links.

Moreover, a closer look to some predicted cases of combinatorial control gives interesting insights into the biology of drug resistance. In particular, we find RPN4, a transcriptional regulator of the proteasome, regulated by both PDR3 and YAP1. This interaction between drug resistance and the proteasome was already hinted in previous works concerning global stress resistance [54], and was recently proved experimentally [1]. This case is not found when running ARACNe on the same dataset, emphasizing the need for specially designed algorithms in order to uncover new cases of combinatorial control. Another interesting case of combinatorial regulation predicted in this analysis is the cross regulation of YAP1 and RAS1 by PDR1, PDR3 and RPN4. This complex regulation could therefore link drug resistance and proteasome regulation to the processes of cell aging and proliferation, regulated by RAS1. However, to our best knowledge there is no experimental evidence of this link, which is to be confirmed.

## Conclusions

In this work, we have presented an effcient method for genome-wide inference of regulatory networks, particularly designed to take into account cases of genetic combinatorial control. The method, based on message passing, was tested on a small *in-silico *model for the cell-cycle regulation in yeast, and then applied to both a large-scale and a small-scale dataset. The test shows the accuracy of the method in case of informative data, and the applications predict meaningful network structures.

One relevant feature of our algorithm is its capability of unveiling patterns of combinatorial control. Even if the model of gene-regulation we used (linear superposition of inputs, followed by a non-linear function) is very simple, it allows for regulators which account only for part of the target expression, and which may be corrected for by other regulators under other conditions, cf. Fig 3.

From the algorithmic point of view, our methodology allows to explore combinatorially the full space of regulatory networks while keeping the computational time short. The flexibility of the approach allows for integrating other type of data: to give an example, information about putative transcription factor binding sites in the regulatory region of an output gene can be easily integrated via a transcription-factor dependent diluting field *h*.

Finally, our method can be generalized to tackle a variety of issues in the field of gene regulation inference. One possibility is to try to discover new regulators, by a corrective methodology, starting with a known regulatory network and looking for the most relevant regulations to be added to this network. Another possibility is to use the information of combinatorial control in conjunction with the nature of the expression data to explain which conditions allow which combinatorial controls, opening the door to a wealth of genetic experiments and to a better understanding of the complexity of gene regulation.

## Methods

### Data encoding

Gene expression data are encoded into a (*N *+ 1) × *M input *matrix of entries , with *i *= 0.1,..., *N *and *μ *= 1,..., *M*, where *M *is the number of experiments (arrays), *N *+ 1 is the number of genes. The value  is a real number that quantifies the level of expression of gene *i *in sample *μ*; more precisely,  is the *i *log-ratio of the actual expression of the gene *i *and the expression of the same gene in a reference condition. A negative (positive) value indicates the under- (over-)expression of gene *i *i sample *μ *with respect to the reference. Here we use the vectorial notation  to indicate expression pattern *μ*.

The task is the reconstruction of a network model which may explain these data. Using a statistical-physics analogy, starting from some snapshots of the microscopic state of a system one tries to infer the energy function (Hamiltonian) governing its behavior. Note that due to the directed nature of gene networks this task can be formally factorized over regulated genes: we can ask first, which genes have a regulatory influence on gene 0, and how they interact combinatorially. Then we ask the same question for the regulators of gene 1, 2,..., *N*. To further simplify the possible influence other genes can have on target gene 0, we aim at a ternary classification of the influence of a gene *i *on 0:

This classification scheme is clearly an oversimplification with respect to biological reality, where a whole range of positive and negative interaction strengths is expected. On the other hand, given the peculiar restriction posed by the limited number of available expression patterns, having a simple but meaningful model reduces the risk of overfitting and produces results which are easier to interpret. Our algorithm can be easily extended to include more than three values for the *J*_*i*→0_; in most cases we have analyzed this generalization does not increase the predictive power.

### A minimal deterministic model of gene regulation

As a minimal functional model, we assume that a gene becomes over-expressed if the joint influence of its regulators is above some threshold -τ, and it is repressed if the joint influence is below -τ. Hence, indicating the sign function by sign(·), and introducing , we expect(3)

to hold for as many expression patterns *μ *= 1,..., *M *as possible. In this sense, each pattern poses a *constraint *on the coupling vector **J **= (*J*_1→0_,..., *J*_*N*→0_), and the problem of finding a good candidate vector **J **can be understood as an instance of a *constraint satisfaction problem*. A cost function for this problem counts the number of errors made in Equation (3),(4)

with Θ being the Heaviside step function. Obviously threshold functions form only a restricted function space. Functions like a XOR (or real-valued generalizations of it) are not implementable in this way. However, due to the before-mentioned problems with data quantity and quality and the risk of overfitting we must restrict our model to a class of functions which is biologically reasonable but does not depend on too many parameters.

The simplest prior biological knowledge we can include at this point is the *sparsity *of regulatory networks. In this sense, we are looking for coupling vectors **J **which are as sparse as possible, *i.e*. which contain as few as possible non-zero elements. The number of these entries is counted by(5)

and will be incorporated into the cost function,(6)

with  acting as a parameter controlling the relative importance of the two contributions: A small *h *will favor couplings of low ℋ_0_, a high *h *will force couplings to be sparse at the cost of possibly not satisfying some pattern constraints. We introduce a formal inverse temperature *β *and the corresponding Gibbs distribution(7)

with . At the end we are interested in the low-*β *case where the Gibbs distribution concentrates in low-cost configurations.

### A minimal stochastic model of gene regulation

The previous deterministic scheme is appealing for its simplicity but does not take into account the noise present in real data. We will assume first that the actual expression value of gene 0 is given as the sum of the measured value  and a Gaussian noise *η *of zero mean and variance σ^2^:(8)

Denoting a centered Gaussian of variance σ^2 ^by , we can write the probability of measuring a given value  for variable 0 as(9)

For σ → 0 we go back to the deterministic model (3), σ > 0 smoothes the Heaviside function into a sigmoidal function. The noise level that we estimate from data is encoded in the value of σ.

### Bayesian Inference

We turn (9) into a probabilistic Bayesian framework [55]. Assuming statistical independence of x^*μ *^for *μ *= 1,..., *N*, Bayes theorem allows to write the posterior probability of a coupling vector **J**:(10)

As a prior for the coupling we use the distribution *P*(**J**) ∝ exp{-*hN*_eff_(**J**)} favoring (sparse) connections with small *N*_eff_. Unsurprisingly, for σ → 0 one recovers Equation (7).

From this point of view the choice of the prior is analogous ℒ_1 _regularization method [56], but on a discrete vector of elements in {±1, 0}, *i.e. *in a case where the ℒ_1 _regularization is equivalent to the ℒ_0 _one.

### Belief Propagation

The belief propagation (BP) algorithm is exact on tree-like graphical models, but it has been extensively used as an heuristic procedure to solve problems defined on sparse graphs [57,58]. Recently, the same approach has been shown to be a good approximation also for problems with dense graph structure [59-61]. BP is an iterative algorithm for estimating marginal probability distributions. It works by locally exchanging messages, until global consistence is achieved. The messages sent between variable nodes *i *(couplings) and function nodes *μ *(constraints) are:

• The probability *ρ*_*μ*→*i*_(*J*_*i*→0_) that constraint *μ *forces variable *i *to assume value *J*_*i *→ 0_.

• The probability *P*_*i*→*μ*_(*J*_*i*→0_) that variable *i *takes value *J*_*i*→0 _in the absence of constraint *μ*.

The BP equations establish an approximate relation between these messages,(11)(12)

Proportionality constants are easily determined by normalization. The algorithm starts from randomly initialized messages and stops when convergence is reached. Our convergence criterion requires the difference between all message at time *t *and the corresponding ones at time *t *- 1 to be less than a pre-defined threshold (10^-8 ^in our simulations). Upon convergence marginal probability distributions are given by(13)

From the point of view of algorithmic complexity, Eq. (12) still contains a sum over (3^*N*^) terms, so the direct implementation of BP is not feasible for large systems. This problem can be solved approximately: Eq. (12) can be understood as the average of  over *N *- 1 independent random variables {*J*_*j*→0_|*j *≠ *i*}, with  depending on the coupling vector only via the sum . For a sufficiently large system we can use the central limit theorem and approximate the exponential sum by a single Gaussian integration,(14)

With(15)(16)

The notation ⟨·⟩_*j*→*μ *_stands for the average over P_*j*→*μ*_(*J*_*j*→0_).

Of course the central limit theorem is meant to be valid in the limit of *N *→ ∞. In practice the Gaussian approximation produces the same results as the exact computation of Eq. (12) already for *N *~ 10 (where the exact computation is clearly feasible).

### Computational complexity

By means of the Gaussian approximation, the complexity of Eq. (12) is reduced from (3^*N*^) to (*N*), and that of the overall iteration to (*MN*). The apparent complexity (*MN*^2^) of updating *M N *messages in time (*N*) can be reduced to (*MN*). by a simple trick: The sums in Eqs. (16) can be calculated over all *j *once for each *μ*, so only the contribution of *i *has to be removed in the update of ρ_*μ*→*i *_for each *i*. This allows to make the single update step in constant time. A precise estimate of the overall complexity of the algorithm would require to control the scaling of the number of iterations needed for convergence. A theoretical analysis of BP convergence times in a general setting remains elusive. Some recent progress for the simpler matching problem can be found in [62]. In all the simulations presented in this work, convergence is always reached in less then 50 iterations.

It would be interesting to compare the efficiency of our algorithm with the computational strategy proposed in [33], based on a Monte Carlo Markov Chain (MCMC) sampler over the model space. In our experience, however, MCMC methods have in general some intrinsically associated problems, mainly due to the fact that the convergence (or mixing) time is hard to assess and often is exponential.

### Observables

*Marginals *- We do not aim at constructing a single high-scoring coupling vector **J **like in a max-likelihood approach. Depending on the shape of the probability space, this vector might be very different from the one actually generating the data. We are instead interested in characterizing the *ensemble *of all high-scoring vectors, or more precisely in the marginal probabilities , which tell us how frequently the coupling from *i *to 0 takes value *J*_*i*→0_. We can therefore base a global ranking of all potential couplings *i *→ 0 on the probabilities 1 - *P*_*i*_(*J*_*i*→0 _= 0) of being non-zero.

When dealing with an artificial data set generated by a known coupling vector **J**^true^, we can measure the similarity of our inference result **J **with the *true *coupling. To this aim we define(17)

The objective of inference is predicting a *fraction *of all couplings with high precision, i.e. to have an as high as possible number of TP with a low number of FP. The quality of the inference can be accounted for by confronting *recall *(or *sensitivity*) *RC *= *N*_*TP *_/(*N*_TP _+ *N*_FN_) and *precision *(or *specificity*) *PR *= *N*_*TP *_/(*N*_*TP *_+ *N*_*FP*_). The recall describes the fraction of all existing non-zero couplings which are recovered by the algorithm, whereas the precision tells us the fraction of all predicted links being actually present in the data generator.

*Entropy *- An interesting quantity to measure is the entropy, *i.e*. the logarithm of the number of high-scoring coupling vectors compatible with our data set. Within BP, it can be approximated by the Bethe-entropy(18)

where *S*^*μ *^= -∑_**J **_*P*_*μ*_(**J**) ln *P*_*μ*_(**J**), and *S*_*i *_= -∑_*Ji *_P_*i*_(*J*_*i*_) ln *P*_*i*_(*J*_*i*_), and *P*_*μ*_(**J**) is defined as(19)

*i.e*. it takes into account the contribution of a single constraint to the probability distribution of **J**.

### Parameter fixing and zero-entropy criterion

The diluting field *h *is the conjugate variable of the number of effective link , so we can equivalently fix one of the two quantities. One can decide to fix the number of effective links, and thus the size of the searched gene signature, and to choose *h *accordingly. To find the correct value of *h *we apply a cooling procedure where, after each interaction of the BP equations step, we increase (resp. decrease) *h *depending on whether the effective number of link is higher (resp. lower) than the desired value. Since the true number of relevant genes is an unknown quantity, the chosen value for , itself is a free parameter. In practice, in the cooling procedure of the *h *field, we monitor the value of the entropy and we stop the iteration when as soon as it becomes lower then zero, *i.e*. at the point where we are able to restrict the of the number of possible solution to our problem to a sub-exponential number (remember that the entropy here indicates the logarithm of the number of solutions). Upon a further increase of *h *the entropy becomes negative, and no zero energy solution is found at that value of the dilution parameter *h*.

In all our simulations we have taken the limit *β *→ ∞.

## Authors' contributions

All authors equally contributed to this work. All authors read and approved the final manuscript.

## Supplementary Material

Additional file 1 All files are in additional file 1. Click here for file
